# Long-read sequencing reveals extensive gut phageome structural variations driven by genetic exchange with bacterial hosts

**DOI:** 10.1126/sciadv.adn3316

**Published:** 2024-08-14

**Authors:** Senying Lai, Huarui Wang, Peer Bork, Wei-Hua Chen, Xing-Ming Zhao

**Affiliations:** ^1^ Department of Neurology, Zhongshan Hospital and Institute of Science and Technology for Brain-Inspired Intelligence, Fudan University, Shanghai, China.; ^2^ State Key Laboratory of Medical Neurobiology, Institutes of Brain Science, Fudan University, Shanghai, China.; ^3^ MOE Key Laboratory of Computational Neuroscience and Brain-Inspired Intelligence, and MOE Frontiers Center for Brain Science, Fudan University, Shanghai, China.; ^4^ Key Laboratory of Molecular Biophysics of the Ministry of Education, Hubei Key Laboratory of Bioinformatics and Molecular Imaging, Center for Artificial Intelligence Biology, Department of Bioinformatics and Systems Biology, College of Life Science and Technology, Huazhong University of Science and Technology, Wuhan, Hubei, China.; ^5^ European Molecular Biology Laboratory, Structural and Computational Biology Unit, Heidelberg, Germany.; ^6^ Max Delbrück Centre for Molecular Medicine, Berlin, Germany.; ^7^ Department of Bioinformatics, Biocenter, University of Würzburg, Würzburg, Germany.; ^8^ College of Life Science, Henan Normal University, Xinxiang, Henan, China.

## Abstract

Genetic variations are instrumental for unraveling phage evolution and deciphering their functional implications. Here, we explore the underlying fine-scale genetic variations in the gut phageome, especially structural variations (SVs). By using virome-enriched long-read metagenomic sequencing across 91 individuals, we identified a total of 14,438 nonredundant phage SVs and revealed their prevalence within the human gut phageome. These SVs are mainly enriched in genes involved in recombination, DNA methylation, and antibiotic resistance. Notably, a substantial fraction of phage SV sequences share close homology with bacterial fragments, with most SVs enriched for horizontal gene transfer (HGT) mechanism. Further investigations showed that these SV sequences were genetic exchanged between specific phage-bacteria pairs, particularly between phages and their respective bacterial hosts. Temperate phages exhibit a higher frequency of genetic exchange with bacterial chromosomes and then virulent phages. Collectively, our findings provide insights into the genetic landscape of the human gut phageome.

## Teaser

The landscape of structural variations across the human gut phageome is revealed via long-read metagenomic sequencing.

## INTRODUCTION

Phages, including bacteriophage and archaeal viruses, are ubiquitous in the human gut (*1*) and have pivotal roles in modulating the bacterial community (bacteriome) and facilitating horizontal gene transfer (HGT) among bacteria (*2*, *3*). The human gut phage community (phageome) primarily comprises tailed double-stranded DNA phages (dsDNA phages), including the prevalent *crAssphage* and *Gubaphage* (*4*, *5*). Recent metagenomic studies have revealed high interindividual taxonomic variation of gut phageome structure (*1*, *6*). Apart from taxonomic variation, the fast-evolving nature of phage genomes, which manifests as high genetic variation within the same phage species, is also a vital contributor to the high interpersonal diversity of gut phageome (*7*). Phage microdiversity plays essential roles in phage ecology, and its detection is crucial for understanding adaptation, evolution, as well as phage-bacteria interaction dynamics (*8*).

Both single-nucleotide variations (SNVs) and structural variations (SVs) are major contributors to phage microdiversity (*9*, *10*). Previous investigations into phage microdiversity have shown a progressive accumulation of SNVs in the majority of phage contigs over time (*7*). Moreover, marked microheterogeneity was detected within phage populations, whereby each phage type was represented by a composite of multiple phage strains at any particular time point (*11*). Despite those findings, previous studies focused mainly on subtle genetic variations based on short-read sequencing, while the larger genetic variance, specifically SVs, remains unexplored.

SVs are variable genomic segments that could harbor functional genes underling phenotypic characteristics, such as virulence, host immune evasion, and pathogenicity (*12*, *13*). Unlike single-nucleotide polymorphisms, which alter a single nucleotide, SVs encompass larger genomic alterations and can result in more substantial functional changes, potentially providing a rapid means for phages to adapt to new hosts and escape host defenses (*14*). Thus, they offer a subgenome resolution of phage functionality and may play more notable roles than SNVs. As a result, profiling the gut phageome in terms of SVs is urgently needed to better understand phage evolution and diversification. Such profiling can be facilitated by long-read sequencing technologies, such as Pacific Biosciences (PacBio) or Oxford Nanopore Technologies, which offer a great advantage in SV detection due to their ability to cover large genomic regions (*15*).

Here, we present a comprehensive characterization of SVs in the human gut phageome with the utilization of viral-like particles (VLPs) enriched long-read sequencing across 91 individuals. As a result, 14,438 nonredundant phage SVs have been identified in 9401 metagenome-assembled high-quality gut DNA phages. We found that these phage SVs were enriched in recombinases, bacteria-derived functions, and antibiotic resistance genes. The functional repertoire harbored by SVs showed distinct characteristics in relation to phage lifestyles. Intriguingly, the formation of the majority of phage SVs was driven by phage-host genetic exchange, as evidenced by their homology to bacterial host fragments and the enrichment of these SVs in bacteria-to-phage (B-to-P) transferred genes. Temperate phages exhibited a heightened proclivity for genetic exchange with bacterial hosts, resulting in a higher SV density compared to virulent phages. Overall, our results reveal functional capacities of phage SVs and facilitate insights into the genetic variance of human gut phageome.

## RESULTS

### SVs are prevalent in the human gut phageome

To systematically characterize SVs within the human gut phageome, we explored our recently constructed Chinese Human Gut Virome (CHGV) that contains 91 virus-enriched PacBio sequencing (vPBS) fecal samples, along with matching virus-enriched short-read sequencing [VLP-enriched next-generation sequencing (vNGS)] and bulk metagenomic samples (without virome enrichment) (*16*). The established CHGV catalog consists of 21,648 nonredundant phage genomes that were built with hybrid assembly using both PacBio long reads and Illumina short reads. We focused on the subset of 9401 phage genomes with >90% completeness estimated by CheckV (*17*), referred to as CHGV-HQ (for “high quality”).

**Fig. 1.**
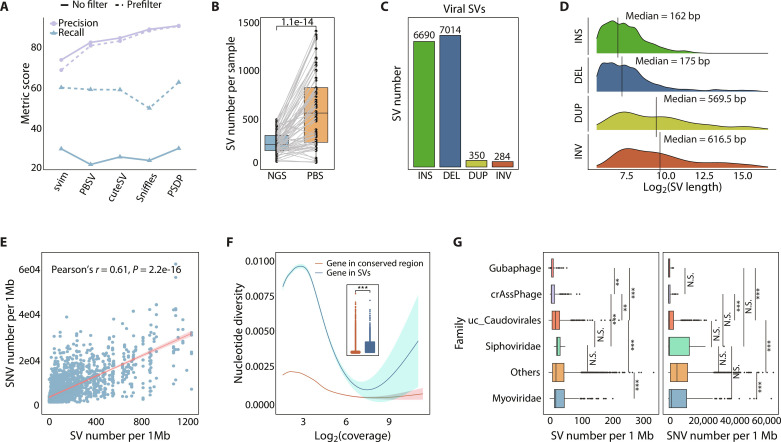
Characterization of SVs in the human gut phageome with PacBio long reads. (**A**) Evaluation of long-read–based SV detection algorithms over simulated virome enriched metagenomic data. “Prefilter” refers to phage genomes from the CHGV-HQ catalog that are filtered with sequence identity threshold of 0.90, while “no filter” refers to those phage genomes without filtering. (**B**) The number of phage SVs detected by Illumina short reads (NGS) and PacBio long reads (PBS), where the *P* values obtained with paired *t* test are shown. (**C**) The number of detected nonredundant INSs, DELs, INVs, and DUPs for 9401 high-quality phages. (**D**) The length distribution of each SV type with the black line indicating the median length of each SV type. (**E**) Positive correlations between the average number of SVs and SNVs per 1 Mb viral genome. The red line represents the regression line with shaded region showing 95% confidence interval. (**F**) Comparison of nucleotide diversity of genes residing within phage SVs and conserved regions at varying read coverage. The inner boxplot shows the overall comparison of nucleotide diversity of genes located in conserved regions and phage SVs. (**G**) The family-wise distribution of the number of phage SVs (left) and SNVs (right) per 1 Mb genome [SV: *P* < 2.2 × 10^−16^; SNV: *P* < 2.2 × 10^−^^16^; analysis of variance (ANOVA) test]. Asterisks represent statistical significance of multiple testing corrected two-sided Mann-Whitney *U* test [**false discovery rate (FDR) < 0.01, ***FDR < 0.001]. In boxplots, boxes span from the first to the third quantiles and black horizontal lines represent the median, with whiskers extending 1.5 times the interquartile range (IQR). N.S., not significant.

The PacBio reads corrected with circular consensus sequencing (CCS) were mapped to the CHGV-HQ genomes to detect four types of SVs: insertions (INSs), deletions (DELs), inversions (INVs), and duplications (DUPs). Specifically, we built a phage SV detection pipeline (PSDP) by combining four well-established SV callers specifically designed for long-read mapping-based SV discovery, namely, Sniffles (*18*), SVIM (*19*), pbsv, and cuteSV (*20*) (see Materials and Methods). To ensure read alignment accuracy, we implemented a prefiltering step for each sample by retaining phage genomes from the CHGV-HQ catalog that exhibited a minimum sequence identity of 0.90 with the sample. As a result, a total of 9183 phage genomes were retained. Benchmarking on simulated long-read metagenomic datasets that were generated with CHGV-HQ phage genomes with Metagenome read simulation of multiple synthetic communities (MGSIM) simulator (Materials and Methods) (*21*), PSDP outperformed all other SV callers, achieving a precision of ~91% (Fig. 1A and fig. S1A). Notably, the prefiltering step substantially improved recall with comparable precision compared to the results without a filtering step (Fig. 1A and fig. S1A).

PSDP was then applied to our vPBS sequencing fecal samples from 91 individuals. An average of 490 phage SVs per individual was identified, with DELs and INSs constituting 52 and 43% of the total, respectively. To construct nonredundant SVs, we merged detected SVs across all samples for each SV type (see Materials and Methods), resulting in a total of 14,438 nonredundant phage SVs (6690 INSs, 7014 DELs, 350 DUPs, and 284 INVs; Fig. 1C). An average of seven SVs were detected per phage genome (fig. S2A). Notably, the detected number of SVs per phage genome exhibited a significant correlation with the read coverage (Spearman’s *r* = 0.38, *P* < 2.2 × 10^−16^; fig. S2B), indicating that sufficient sequencing depth is required to comprehensively characterize SVs. The median lengths of INSs and DELs were 162 and 175 bp, respectively, which were considerably shorter than those of DUPs (569.5 bp) and INVs (616.5 bp; Fig. 1D). Manual inspection of a random subset of 50 nonredundant SVs using IGV (*22*) confirmed that approximately 90% of them were supported by more than one PacBio reads, thus confirming the confidence of our detected phage SVs (figs. S2C and S3 and Materials and Methods).

We further explored the relationship between SNVs and SVs in phages, whereby SNVs were identified by mapping vNGS reads onto the CHGV-HQ genomes given their high base accuracy. The number of SVs per 1 Mb (referred to as “SV density”) in phage genomes strongly correlated with the SNV density (Pearson’s *r* = 0.61, *P* < 2.2 × 10^−16^; Fig. 1E). Furthermore, the genes located within SV regions typically had significantly higher nucleotide diversity than those in conserved regions, indicating that these genes may be under distinct selective pressure (Fig. 1F). Comparison of nucleotide and structural microdiversity across phage clades showed that both SNVs and SVs have strong family-specific density (Fig. 1G), similar to the previously discovered uneven distribution of SV frequency across bacterial taxonomic groups (*23*). Together, by using long reads, we identified a substantial number of SVs and illustrated their prevalence in the gut phageome (Fig. 1B).

### Phage SVs are enriched with mobile element–associated and bacteria-derived functions

**Fig. 2.**
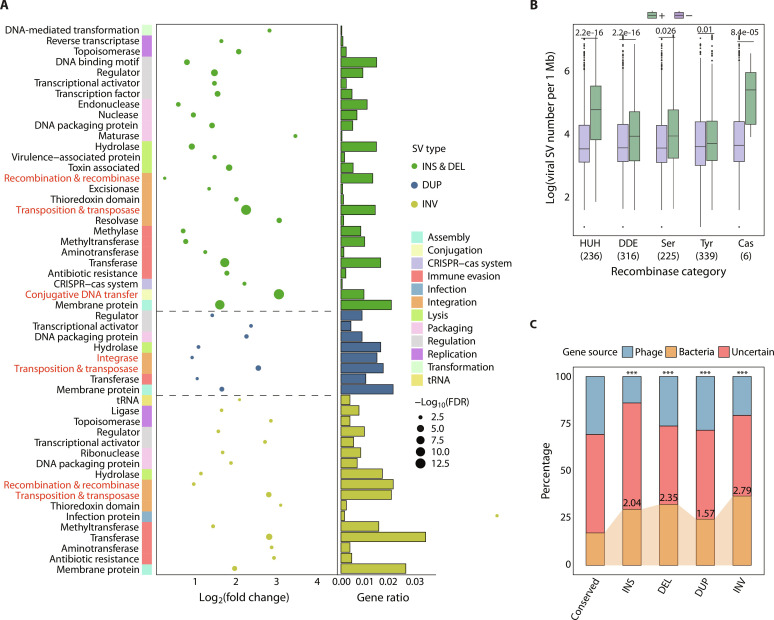
Enriched functions and sources of various phage SVs in the human gut phageome. (**A**) The enriched functional categories of phage SVs for each SV type (one-sided Fisher’s exact test, FDR < 0.05). Mobile element–associated functions are highlighted in red. tRNA, transfer RNA. (**B**) Boxplots of the number of SVs per 1 Mb between phages with (+) and without (−) a specific type of recombinase (*24*). *P* values of two-sided Mann-Whitney *U* test are shown. The gene count per recombinase category detected within phage SV regions is displayed in bracket along the *x* axis. (**C**) Distributions of the likely sources of the phage genes stratified by their locations in four SV types (i.e., INS, DEL, DUP, and INV) and conserved regions (genomic regions without SVs). Asterisks represent statistical significance of Fisher’s exact test (***P < 0.001), and the values of odds ratio relative to the conserved are shown.

Phage SVs may carry functional genes or introduce breaking points in coding regions, thereby leading to strain-level phenotypic and functional differences. Thus, we searched for gene-coding regions that overlapped with phage SVs and annotated the functions associated with these SVs. We found a significant overrepresentation of genes related to mobile element–associated functions, such as recombinases and transposases, across all types of SVs (Fig. 2A), with approximately 8% of SVs carrying recombinases or transposases.

Using 68 previously (*24*) described calibrated hidden Markov model (HMM) profiles, we classified the recombinases into five major families: HUH, DDE, serine (Ser), tyrosine (Tyr), and cas1 (Cas) recombinases. The most prevalent recombinase family within phage SV regions was the Tyr recombinase, consisting primarily of the prophage integrase family (PF00589: phage_integrase), followed by the DDE recombinases (or transposases) actively found in IS elements and responsible for genetic transposition (Fig. 2B) (*25*). Comparing the SV density between phage genomes with and without a particular type of recombinase, we observed that phages harboring recombinases generally exhibited higher SV density than recombinase-deficient ones (*P* < 0.05, two-sided Mann-Whitney *U* test; Fig. 2B). Moreover, we revealed an enrichment of phage SVs with genes associated with antibiotic resistance, DNA methylation, and toxin-antitoxin systems (Fig. 2A), the presence of which has been implicated in evading host immunity and conferring survival fitness for phages (*26*).

Phage SVs were additionally enriched with several bacteria-derived functions, such as DNA transformation, conjugation systems, and CRISPR-Cas systems, underscoring the potential active participation of bacteria in the formation of phage SVs. To ascertain the enrichment of bacterial genes within phage SVs, we annotated the gene sources as either bacterial or phage using a built-in CheckV database (*17*). As expected, we found a higher proportion of bacteria-derived genes within phage SV-containing regions, compared to genes predicted in “conserved” regions (genomic regions lacking SVs) (*P* < 2.2 × 10^−16^, Fisher’s exact test; Fig. 2C). Therefore, the presence of bacterial genes within phage SVs implies the occurrence of B-to-P gene transfers.

### B-to-P HGT events are involved in the formation of phage SVs

**Fig. 3.**
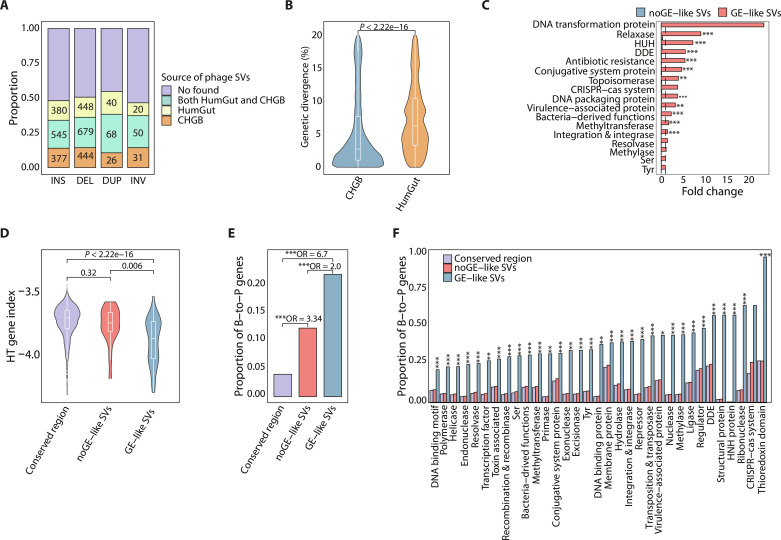
Phage-bacteria genetic exchange as a driving force for the formation of phage SVs. (**A**) Prevalence of phage SVs that have high homology to bacterial fragments in each SV category. (**B**) Genetic divergence between GE-like phage SV sequences and bacterial fragments derived from CHGB and HumGut (*27*) datasets, respectively. (**C**) The enrichment of genes related to genetic exchange in regions with noGE-like phage SVs and GE-like phage SVs, respectively (one-sided Fisher’s exact test, **FDR < 0.05, ***FDR < 0.001). (**D**) The HT gene index of genes in conserved region, regions with noGE-like SVs and GE-like SVs. *P* values of two-sided Mann-Whitney *U* test are shown. (**E**) Comparison of the proportion of B-to-P transferred genes in conserved region, regions with noGE-like SVs and GE-like SVs (Fisher’s exact test, ***FDR < 1 × 10^−^^05^). (**F**) Comparison of the proportion of B-to-P transferred genes in conserved region, regions with noGE-like SVs and GE-like SVs, stratified by different functional categories (Fisher’s exact test, *FDR < 0.05, **FDR < 0.01, and ***FDR < 0.001). OR, odds ratio.

The enrichment of mobile element–associated functions and bacteria-derived genes in phage SVs prompted an investigation into the role of phage-bacteria genetic exchange in phage genomic diversification. For this, we looked for homologs (>80% identity, covering >80% of the query sequences) of phage SV sequences longer than 200 bp (6273 SVs) in the bacterial genomes from two datasets: Chinese Human Gut Bacterial Catalog (CHGB) and HumGut (*27*). The CHGB dataset encompasses metagenomic-assembled genomes derived from paired bulk metagenomic data (i.e., no virome enrichment) of the CHGV cohort (see Materials and Methods), while the HumGut dataset (*27*) is a public database that contains a comprehensive collection of gut bacterial genomes. Notably, 3108 phage SV sequences (49.5%) showed homology to bacterial fragments (Fig. 3A and fig. S4), suggesting that these SV sequences potentially resulted from phage-bacteria genetic exchange events. We hereafter referred to these SV sequences as “GE (genetic exchange)–like SVs,” while the remaining were categorized as “noGE-like SVs.”

Among the GE-like phage SVs, the majority (71%; 2220 SVs) had matches with the CHGB genomes obtained from the same cohort (Fig. 3A). Despite 43% (1342 SVs) of them exhibited alignment with both the CHGB and HumGut genomes, we observed that these SV sequences displayed significantly lower genetic divergence from the CHGB genomes compared to those from the HumGut dataset (Fig. 3B). This finding suggests a recent acquisition of GE-like SV sequences from the CHGB genomes rather than the HumGut genomes, potentially explained by the fact that HGT mostly occurs between genomes inhabiting the same environment (*28*) and is often transient.

To further investigate whether these GE-like SVs resulted from HGT events, we compared the functions associated with GE-like SVs and noGE-like SVs. We looked at the dissimilarity between GE-like SV sequences and their genomic background using a horizontally transferred gene index (HT index; see Materials and Methods) (*29*); a lower HT index indicates a greater degree of dissimilarity, and the regions with low homology to their genomic context are indicative of genetic material acquired from other genomes and often associated with the occurrence HGT events (*29*). As expected, the GE-like SVs exhibited the highest dissimilarity compared to other regions of the same genomes (Fig. 3D), supporting their origins from different genomes. As expected, known HGT-related genes, particularly the DDE and HUH recombinases, along with other HGT-related genes including relaxase and integrase were significantly enriched in the GE-like SVs instead of noGE-like SVs (Fig. 3C and fig. S5). In addition, ~42% of these GE-like SVs (1341 SVs) were found to carry bacteria-derived genes (inferred from CheckV annotations), further confirming that they were shaped by HGT events.

To ascertain the transferred directionality of GE-like SVs, we used a molecular phylogenetic-based approach to find HGT-indicative topologies within evolutionary trees, a robust method for determining HGT directionality (Materials and Methods) (*30*). We identified a total of 33,572 B-to-P and 79,033 phage-to-bacteria (P-to-B) HGT events. The B-to-P events, rather than P-to-B events, were significantly enriched in GE-like SV-containing regions (Fig. 3, E and F), supporting the bacterial origin of these GE-like phage SVs. We observed that the majority of recombinases and transposases located within GE-like SV regions were derived from B-to-P HGT events, with approximately 60% of DDEs carried by GE-like SVs identified as B-to-P HGT genes (Fig. 3F).

These genetic transfer events have the potential to facilitate the dissemination of microbial traits across kingdoms. For instance, 46% of methylases and 33% of methyltransferases located within GE-like SV regions were attributed to B-to-P HGT events (Fig. 3F). Both methylases and methyltransferases can serve as a defense mechanism against host bacterial restriction modification system, thereby enhancing the viability advantages conferred to phages (*31*). In addition, we identified several instances of B-to-P genetic transfer events involving CRISPR-associated cas1 proteins, implying the acquisition of phage-encoded CRISPR systems from bacteria (fig. S6). Collectively, these findings confirm the involvement of B-to-P genetic exchange in the formation of phage SVs.

### The genetic exchange of GE-like SV sequences between specific phage-bacteria pairs

**Fig. 4.**
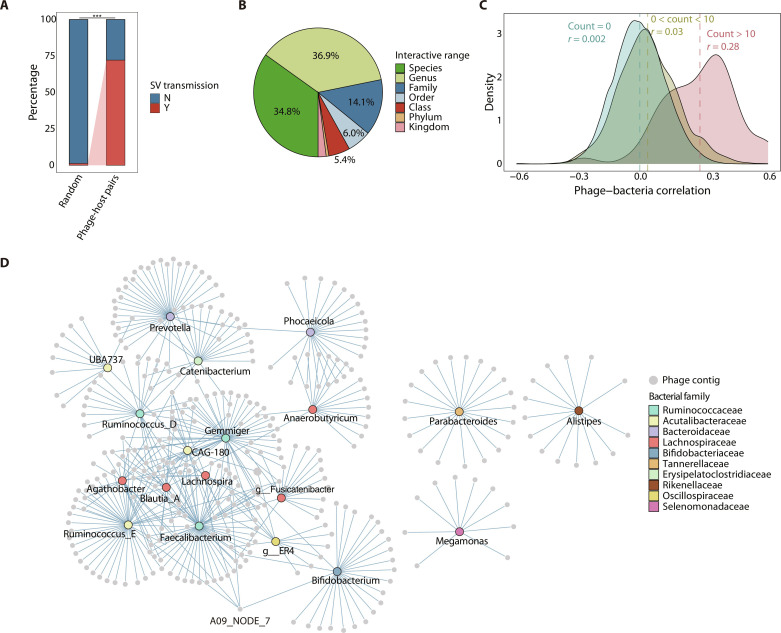
The transmission of GE-like SV sequences between bacteria and phages. (**A**) Comparison of the percentage of GE-like SV sequence transmission that occurred within inferred phage-host pairs and random phage-host pairs (non-PH pairs). Statistical significance of Fisher’s exact test is shown (***P < 2.2 × 10^−16^, odds ratio = 212.2). (**B**) The proportion of the CHGV-HQ phages estimated using the GE-like pSVs (phage SVs) at different interactive ranges. Here, the “range” refers to the taxonomic rank of the last common ancestor of bacterial genomes that have BLAST matches to GE-like SV sequences. (**C**) Distribution of phage-bacteria correlations across the 91 individuals, stratified by the phage-bacteria pairs with different number of shared GE-like SVs, where count = 0 means the number of pairs of phage and bacteria without any shared SVs, 0 < count < 10 means the number of pairs of phage and bacteria sharing less than 10 SVs and the same for count >10. The dashed lines show the median correlation in each distribution. (**D**) Phage-bacteria interaction network with edges indicating that there are shared GE-like phage SVs between phages and bacteria.

As phages typically have a narrow host range and undergo coevolution with their bacterial hosts (*32*, *33*), we postulated that the presence of GE-like SVs arises from phage-host interactions. We determined the phage-host relationships based on CRISPR spacer similarity (Materials and Methods) (*34*) and subsequently investigated whether the phage GE-like SV sequences exhibit homology with genomic fragments derived from their predicted bacterial hosts. We noticed a significantly higher incidence of GE-like SV sequence homology observed within inferred phage-host pairs (~73%; Fig. 4A), suggesting potential sequence transmission between them. While a single phage contig could have multiple GE-like SVs, our findings indicate that these GE-like SV sequences within the same phage contig are predominantly linked to specific bacterial genera (37%) or species (35%, Fig. 4B), consistent with the limited host range of phages. Phage contigs displaying a broader host range exhibited a higher SV density (fig. S7A). We further examined the phage-bacteria Pearson’s correlation to understand the interactions between gut phages and bacteria, which is determined on the basis of the abundance of each pair of phage and bacteria at the genus level across the cohort of 91 individuals (Fig. 4C). We noted that phage-bacteria pairs sharing GE-like SVs displayed significant positive correlations (Fig. 4C), and the strength of these phage-bacteria correlations increased with the number of shared SV sequences between them (Pearson’s *r* = 0.37, *P* < 2.2 × 10^−16^; fig. S7B), further implicating the pivotal role of phage-host interactions in phage evolution.

We then analyzed the transmission patterns of GE-like SVs by constructing a phage-bacteria SV sequence sharing network (Fig. 4D). Consistent with the many-to-one relationship observed in phage-host interactions (*35*), multiple phage species acquired genomic fragments from a restricted set of bacteria to form SVs, while a few phages could receive DNA sequences from a broad range of hosts (Fig. 4D). For instance, the phage contig “A09_NODE_7” interacted with more than one bacterial family, including *Acutualibacteraceae* and *Ruminococcaceae* (Fig. 4D). We observed that a large number of phage species were associated with *Faecalibacterium*, a member of the *Ruminococcaceae* family (Fig. 4D), which is known to be one of the most prevalent bacterial genera in the human gut (*3*). It should be noted that the presence of shared GE-like SV sequence between bacteria and phages does not necessarily imply direct genetic transfer between them. Rather, it is more plausible that these shared sequences are facilitated by intermediary genomes or other mobile genetic elements, which we collectively refer to as a “shared mobile element pool” (*28*). For example, phage A may acquire genetic fragments from phage B, which, in turn, obtained those fragments from bacteria. Therefore, while there is no direct genetic transfer between phage A and bacteria, there are indirect interactions mediated by phage B.

**Fig. 5.**
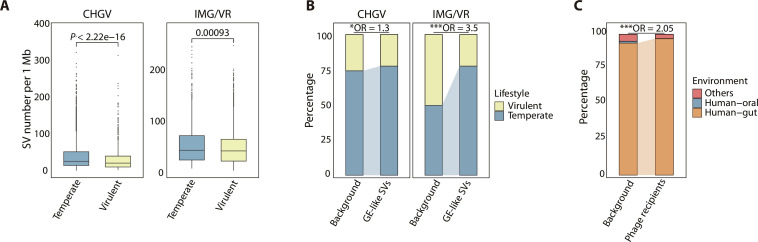
Effects of different lifestyles on phage SVs. (**A**) Comparison of the SV density (number of phage SVs per 1 Mb genome) between temperate and virulent phages in the CHGV-HQ and IMG/VR viral datasets. *P* values of two-sided Mann-Whitney *U* test are shown. (**B**) Enrichment of GE-like phage SVs in temperate phages across CHGV-HQ and IMG/VR viral datasets (*P < 0.05, **P < 0.01, and ***P < 0.001; Fisher’s exact test). “Background” represents the initial proportion of phages with different lifestyles within the respective viral datasets. (**C**) Environmental preferences of phage recipients in the IMG/VR viral dataset. “Phage recipients” represents the phages containing GE-like SVs that are capable of aligning onto bacterial genomes from HumGut and CHGB datasets. Fisher’s exact test was carried out using all phage genomes in the IMG/VR dataset as background (***P < 0.001; Materials and Methods).

As previous studies showed that phages with different lifestyles exhibit distinct mechanisms of phage-host interactions (*36*), we compared the genomic diversity between phages with distinct lifestyles (virulent versus temperate) and noticed that temperate phages harbored significantly higher SV density than virulent phages (Fig. 5A). Both phage lifestyles showed higher proportion of bacteria-derived genes within phage SV-containing regions (fig. S8). However, the prevalence of bacterial genes within the SVs of temperate phages was significantly higher compared to that in virulent phages (*P* value < 2.2 × 10^−16^, odds ratio = 2.31, Fisher’s exact test; temperate phages: 34.1%; virulent phages: 18.3%; fig. S8). The GE-like SVs were also found to be enriched in temperate phages (Fig. 5B), suggesting that phages with a temperate lifestyle undergo more frequent genetic exchange with bacterial hosts than virulent phages. This observation could be attributed to the long-term associations that temperate phages form with host cells within a lysogenic cycle, providing ample opportunities for genetic exchange with their host genomes (*10*). In addition, the functional differences of SVs between temperate and virulent phages can also reflect distinct modes of phage-bacterium interactions (fig. S9). Methyltransferases were found to be enriched in SVs linked to temperate phages rather than virulent phages (fig. S9), contributing to the capacity of temperate phages to establish prolonged associations with bacterial hosts during a lysogenic cycle (*31*). Conversely, we revealed a notable enrichment of endonucleases within SVs of virulent phages (fig. S9). Endonucleases play a pivotal role in the lytic life cycle by degrading the host bacterium’s genomic DNA (*37*), consistent with the characteristic lytic life cycle of virulent phages.

Last, we conducted the same analysis on the Integrated Microbial Genomes and Microbiomes/Viral Genomes Resource (IMG/VR) viral dataset and obtained consistent findings that GE-like SV sequences were transferred between specific phage-host pairs, with notable prevalence observed in the temperate phages (Fig. 5, A to C; figs. S10 to 12; and Materials and Methods). Despite the diverse environmental origin of phage genomes within the IMG/VR dataset, we found that the identified GE-like SV sequences were primarily shared among phages and bacteria residing in the same ecological niche, specifically the human gut (Fig. 5C), demonstrating the ecology as an important factor in shaping SVs. Overall, our results emphasize the critical role of phage-host interactions in phage diversification and their relevance to phage evolution.

## DISCUSSION

SVs are important drivers for the adaptation, speciation, and evolution of the gut phage genomes. In this work, we used single-molecule real-time (SMRT) long-read sequencing to systematically genotype SVs across VLPs-enriched metagenomic samples, revealing the prevalence of phage SVs in the human gut virome. 

While long-read–based SV discovery has the potential to increase the scope of detectable SVs compared to short-read–based SV calling, the reliability of detectable SVs is highly dependent on the quality of phage references and accurate read-to-reference alignments, making direct detection of SVs in the gut phageome still a challenge. The inherent complexity of metagenomics often leads to assembly errors and mismapping of reads against references (*38*), resulting in unreliable SV detection. To address these challenges, we combined both vNGS and vPBS reads to reconstruct phage genomes, as previous studies have demonstrated its efficacy in improving the quality of assembled phage genomes (*39*). In addition, we adopted a prefiltering strategy by removing phage references absent from the sample, given that highly accurate read-to-reference alignments can be achieved by performing on the prefiltered reference database (*40*). Nevertheless, mapping-based SV calling may still overlook a considerable number of true SVs due to insufficient sequencing depth.

In this work, the phage-bacteria genetic exchanges have been identified as a principal contributor to the phage SV formation. However, genetic transfer events occurring among phages could also contribute to the formation of phage SVs. Specifically, errors in the DNA packaging process during phage assembly can lead to the incorporation of DNA fragments from other phage genomes of close lineage into new virions (*10*). In some cases, this can result in the formation of mosaic phages that carry genetic material from multiple phage strains (*10*). Gene transfers have been observed between distantly related phages coinfecting the same bacterial cell (*10*). Phage SVs might also stem from a bidirectional genetic exchange between phages and plasmids rather than bacterial chromosome, as evidenced by the detected enrichment of conjugation-related genes within SV regions (Fig. 2A). A previous study revealed that the phage-plasmids, which show characteristics of both plasmids and phages (*41*), could mediate genetic exchange between phages and plasmids (*42*). Moreover, nonbacterial microorganisms (i.e., archaea, eukaryotes, and protists) colonizing the human gut have the potential to serve as hosts for gut viruses (*43*); thus, the formation of viral SVs could also be facilitated by genetic exchange between viruses and these microorganisms. For instance, one study has systematically identified thousands of HGT events between eukaryotes and viruses (*30*). Therefore, a more detailed analysis of the genetic transfer within phages or between phages and other microorganisms is needed to better understand the cause and consequences of phage diversification.

We observed that phage lifestyle exhibited an association with the functional distributions of SVs, indicating potential divergence in evolutionary trajectories. Notably, methyltransferases exhibited higher enrichment within SVs associated with temperate phages as opposed to virulent phages (fig. S9), which is a characteristic feature of temperate phages. A previous study has illuminated the pivotal role of methyltransferases in the life cycle of temperate phages, where they act as key players in aiding phages to elude the host bacterium’s restriction-modification system. This evasion strategy enables temperate phages to persist in a lysogenic life cycle (*31*), maintaining prolonged associations with their host genomes. Consequently, this elevates the likelihood of horizontal genetic exchange between phages and bacterial hosts, resulting in a heightened SV density compared to phages with a virulent lifestyle (Fig. 5, A to C). We also noted a significant enrichment of excisionases and resolvases within in temperate phage SVs (fig. S9). Both excisionases and resolvases are involved in the excision process, leading to the precise removal of phage DNA from the bacterial chromosome (*44*, *45*). This process facilitates the transition of temperate phages from the lysogenic to the lytic cycle. In contrast, for virulent phages, we found that endonucleases were significantly enriched in the SVs. This observation is consistent with the well-established role of endonucleases in the lytic lifestyle of virulent phages (*37*), wherein they play a pivotal role in degrading the host bacterium’s genomic DNA. The degradation step is crucial for the release of newly synthesized phage particles and the subsequent infection of neighboring bacterial hosts (*37*). Therefore, the observed variations in SV function between the temperate and virulent phages underscore the intricate adaptations that underlie their differing life cycles.

In our work, we organized phage proteins into various functional categories manually with keyword searching. Note that such functional annotations of aggregating diverse biological functions into broad categories may affect the detection of some proteins of certain functions. For instance, the tail proteins play fundamental roles in the infection process of phages and display rapid evolution characteristics that enable them to adapt to changes in host environment and evade host immune responses (*46*). The tail proteins play different functional roles, including tail completion, tail terminator, tail fibers, tail tube, and others (*47*). However, our current functional annotations cannot distinguish these diverse types of tail proteins. In the future, we will refine functional annotations to enable the detection of phage proteins of certain functions.

## MATERIALS AND METHODS

### Generation of high-quality CHGV catalog

The CHGV catalog we established previously (*39*) consists of 21,648 nonredundant phage genomes and was assembled by using a hybrid pipeline that combined both short (Illumina) and long (PacBio) reads. Specifically, approximately 500 g of feces was collected from each of the 135 healthy participants from China, followed by VLP enrichment. The virome enrichment protocol from fecal specimens was adapted from the methodology described in (*48*), with was optimized to accommodate the substantial volume of feces collected from each participant. Specifically, 400 to 500 g of fecal matter, previously frozen at −80°C, was processed with 5 liters of Sodium Chloride-Magnesium Sulfate buffer and homogenized, followed by sequential filtration and centrifugation steps. The filtrate was then concentrated and subjected to NaCl addition and poly(ethylene glycol) 80,000 precipitation, culminating in phage particle sedimentation via centrifugation. The sedimented phage particles were resuspended, mixed with chloroform, and then centrifuged to remove the organic layer, followed by deoxyribonuclease I and ribonuclease A treatment to degrade unprotected nucleic acids. The sample was further processed to remove residual chloroform, ensuring optimal conditions for subsequent enzymatic reactions. Nucleic acid extraction was conducted using the HiPure HP DNA Maxi Kit, conforming to the manufacturer’s specifications. The extraction sequence involved proteinase K and SDS lysis, phenol:chloroform:isoamyl alcohol extraction, and purification through a DNA Mini Column. The purified DNA was then eluted, quantified, and stored at −80°C, ready for downstream analyses. This meticulous approach ensures the integrity and purity of the extracted nucleic acids, essential for accurate virome characterization.

Then, fecal DNAs were extracted and short-read sequenced (vNGS) using Illumina HiSeq2000 sequencing (PE150, Novogen, Beijing, China). Of these samples, 91 samples with sufficient quality of viral DNAs were further submitted to the VLP-enriched third-generation sequencing (vTGS) using a PacBio RS II sequencer (Pacific Biosciences, Menlo Park, CA, USA) with SMRT technology and CCS mode.

We removed contaminations of human and bacterial genomes from both the vTGS and vNGS sequencing datasets by aligning clean reads to the Unified Human Gastrointestinal Genome (UHGG) database and the human reference genome (GRCH 38). To mitigate the risk of overestimating contamination, we identified and excluded possible prophage regions within the UHGG genomes using PhageFinder, thus creating a refined dataset termed UHGG-Minus for this analysis. Reads mapped to these genomes were then excluded from further analysis to eliminate potential contaminants.

Subsequently, a hybrid assembly pipeline was used to construct putative phage contigs as described in our previous work (*16*). Specifically, IDBA-UD v1.1.3 (*49*) was used to assemble the filtered vNGS data, using parameters: -maxk 120 -step 10 -min_contig 1000. Meanwhile, Canu v2.0 (*50*) and Flye v2.8.2 (*51*) were used to assemble the filtered vTGS CCS reads. Given Canu’s absence of a meta-assembly mode and its tendency to elongate contigs by amalgamating DNA sequences from distinct phage species, unitigs were prioritized for subsequent analysis. Unitigs, fundamental blocks of contigs, exhibited shorter lengths but greater reliability compared to contigs (*52*). Following this, MetaBAT2 v2 (*53*) was applied to cluster unitigs into bins to further extend sequences, with contigs instead of unitigs being used for further analysis if all unitigs from a single contig could be grouped into the same bin. OPERA-MS v0.9.0 (*54*) and metaSpades v3.13.1 (*55*) were used for hybrid assemblies by incorporating both vTGS and vNGS reads from identical samples. Thereafter, contigs and unitigs obtained through the aforementioned three strategies were consolidated. In case where vTGS reads were unavailable, contigs derived from the IDBA-UD assembler were used.

To obtain nonredundant phage datasets, phage contigs assembled from all samples were merged and then dereplicated using CD-HIT (*56*) with a global identity threshold of 95%. After that, contigs longer than 5 kb or circular ones longer than 1.5 kb were retained and referred to as the CHGV catalog. Last, we selected a high-quality subset of viral genomes from the CHGV catalog (CHGV-HQ) for subsequent analysis. In total, we obtained 9665 near-complete dsDNA phages with >90% completeness, of which the quality of phage genomes was estimated by CheckV (*17*).

### Detection of viral SVs using long reads

Viral SVs were identified for the 9665 high-quality intestinal phage genomes (CHGV-HQ catalog) across 91 vTGS samples with SMRT sequencing data. Initially, we established a prefiltered reference phage database unique to each sample by retaining phage genomes that exhibiting a minimum sequence identity of 0.90 with the respective vTGS sample, as measured by MASH distance (*57*). As a result, an average of 904 phage genomes from the CHGV-HQ catalog was selected as references for each sample. Subsequently, PacBio CCS reads were aligned to the sample-specific reference phage database using PBMM2 (https://github.com/PacificBiosciences/pbmm2), followed by the application of multiple long-read–based SV callers for each sample. Notably, the prefiltering step yielded a significantly greater number of SVs compared to using all CHGV-HQ phage genomes as references for each sample (fig. S1B). To obtain high-confidence phage SVs, we used four SV callers, namely, Sniffles v2.0.6 (*18*), cuteSV v1.0.13 (*20*), pbsv v2.3.0 (https://github.com/PacificBiosciences/pbsv), and SVIM v2.0.0 (*19*). These tools are all specifically designed for long-read–based SV discovery and were used with default settings.

We merged phage SV callsets obtained from the aforementioned four SV callers for each sample using a previously described approach (*58*). Specifically, the Cluster Affinity Search Technique (CAST) algorithm (*59*, *60*) was applied to merge SVs independently for each SV type according to their variant position and length. To accommodate INSs into this algorithm, we defined the end coordinate of an INS as the sum of its start coordinate and its length. Initially, all detected SVs were categorized into non-overlapping groups for each SV type, ensuring that the coordinate positions of SVs within the same SV group did not overlap with those of other groups. Within each group, SVs were represented as nodes in a graph, with edges added between two SVs if they exhibited a minimum mutual overlap at least 80% of their length. Subsequently, the CAST-based clustering algorithm was applied on the corrupted clique graph derived from every SV group. Last, we retained SVs discovered by at least two SV callers for each sample. According to the benchmarking results on the simulated virome-enriched metagenomics, Sniffles showed the best performance, followed by cuteSV, PBSV, and SVIM (Fig. 1A). As a result, the results of Sniffles were prioritized to represent the merged SVs, followed by cuteSV, PBSV, and SVIM. In addition, we retained SVs supported by at least two reads and longer than 50 bp to ensure their reliability. While these stringent criteria may overlook some genuine phage SVs, it ensures that SVs detected in our study are well supported by sufficient sequencing depth. To further reduce the possible false positives, the SVs located within 100 bp of the start/end of phage contigs were removed. We further manually validated these SVs by visualizing PacBio reads mapping onto SV-containing regions using IGV (*22*). Briefly, a subset of 50 SVs was randomly selected for manual inspection to ascertain the presence of long reads supporting the detected SVs in the regions covering and surrounding SVs (fig. S2).

To address redundancy of phage SVs obtained across multiple samples, we further merged SVs from all samples using the CAST-based clustering algorithm. Similar to the SV merging process for individual sample, the most prevalent SVs in the population were selected to represent the nonredundant SVs. As a result, we obtained a set of 14,438 nonredundant viral SVs.

### Benchmarking of long-read SV callers on simulated long-read metagenomics

We evaluated the performance of the above four SV callers as well as their merged results on simulated metagenomics. We randomly selected a subset of 1000 phage genomes from CHGV-HQ catalog and introduced known SVs on these genomes. We simulated four common types of SV including 3000 INSs, 3000 DELs, 1000 DUPs, and 1000 INVs on selected 1000 phage genomes with Sim-it (*61*). The MGSIM (*21*) simulator was then used to generate long-read metagenomics in which 6^5^ PacBio reads were simulated with default parameters. We simulated two scenarios, one in which the abundance of phage genomes was uniformly distributed, and the other in which the abundance of each phage genome was sampled on the basis of a lognormal distribution Lognormal (*2*, *10*). We independently simulated three replicate metagenomes for each scenario. The simulated metagenomes were used to validate above four long-read–based SV callers with two alignment modes: mapping reads to all 9665 references of CHGV-HQ catalog (no filter) or selected 1000 phage genomes (prefilter). We used Truvari (*62*), an SV comparison toolkit, to calculate performance metrics (precision, recall, and *F1*) of all SV callers by comparing the VCF output of each SV caller against the simulated reference SV set.

### Detection of viral SVs using Illumina reads

We used the paired Illumina sequencing datasets to detect viral SVs for the 9665 high-quality intestinal phage genomes across 104 samples. Similar to the long-read–based SV detection, we built a prefiltered reference viral database for each sample and used BWA-MEM v0.7.17 (*63*) to map Illumina reads to these filtered reference genomes to ensure the highly accurate alignment. We applied three short-read–based SV callers, namely, Manta v1.6.0 (*64*), LUMPY v0.2.13 (*65*), and DELLY v1.0.3 (*66*), which were all commonly used for short-read–based SV identification. We merged SV callsets derived from each SV calling tool using CAST algorithm as described above and retained SVs that were supported by at least two tools. Last, we counted the number of SVs detected in each sample.

### SNV detection in viral genomes

The mapping results (i.e., bam files) of Illumina reads (vNGS) onto filtered reference genomes were used as input to inStrain (*67*), which could provide genome- and gene-wide SNV profiles as well as nucleotide diversity based on short-read alignments.

### Taxonomic annotation of phage genomes

Taxonomic annotations of CHGV-HQ phage genomes were achieved using the same method reported in a previous study (*34*). First, phages were assigned to a taxonomic lineage using 21 hallmark phage orthologous gene clusters, which have been recognized as taxon-specific signatures for viral classification (*68*). Second, the phage genomes that clustered with genomes from RefSeq using vConTACT2 v2.0 (*69*) were able to assigned to known taxonomic genera (the reference taxonomy used was the 2018 ICTV Release). Last, we applied a majority rule approach to annotate those phage genomes that could not be assigned to a specific taxonomy with above two steps. The proteins predicted in the phage genomes with Prodigal v2.6.3 (*70*) were aligned against the UniProt database (*71*). At each taxonomic level (up to the genus level), the taxonomy was assigned to a taxon if more than 75% of the proteins are affiliated to this taxon (*72*). Phage lifestyles were annotated using DeePhage v1.0 (*73*) with default parameters.

We further identified crAss-like phages following an approach described in a previous study (*74*). The proteins predicted in phage contigs were aligned to the protein sequences of the polymerase (UGP_018) and the terminase (UGP_092) using blastp (BLAST+ v1.6.2) (*75*), which were genetic signatures of the prototypical crAssphage (p-crAssphage, NC_024711.1). The phage genomes were then classified as crAss-like phages if they had a contig length of at least 70 kb and contained blastp hit with an *E* value < 1 × 10^−5^ of either the p-crAssphage polymerase or terminase. Gubaphages were detected by querying the large terminase protein of the Gubaphage genomes that was obtained from the Gut Phage Database (*76*). The phage genomes containing blastp hit of the large terminase with *E* value < 1 × 10^−5^ were classified as Gubaphages.

### Functional enrichment analysis

The encoded proteins for each phage genome were predicted using prodigal v2.6.3 (*70*) with default parameters. The functions of all viral proteins were annotated by querying against PFAM (*77*), VOGdb (http://vogdb.org/), and eggNOG (*78*) databases using a combination of eggNOG-mapper and HMMER (*E* value < 1 × 10^−5^, score ≥ 50). Annotated genes were functionally classified into following categories (level 1 functional category hereafter) according to a previous study (*79*): packaging, replication, infection, hypothetical protein, regulation, assembly, lysis, integration, immune evasion, transfer RNA, transduction, conjugation, and transformation. For each functional category, we used keyword search to further divide it into more specific categories (level 2 functional category hereafter; table S1). Recombinases were identified by querying all viral proteins against 68 calibrated HMM profiles (*24*) using HMMER (*E* value < 1 × 10^−5^) and were classified into five major families, including HUH, DDE, serine (Ser), tyrosine (Tyr), and cas1 (Cas) recombinases. We additionally identified antibiotic resistance genes by using the ResFinder web portal (*80*). To determine the source of genes, we classified them as bacterial or viral using a built-in database of CheckV, which is composed of 15,958 profile HMMs (*17*). Protein sequences were aligned to these HMM profiles using HMMER with an *E* value threshold of 1 × 10^−5^.

To investigate whether SVs were associated with any particular function, we initially identified genes overlapping with SV regions to link SVs with potential functional groups. The enrichment analysis of specific functions associated with SVs used Fisher’s exact test to compare the frequency of each level 2 functional category in SV-associated genes against those in non-SV regions (conserved regions). We determined significantly enriched functional categories using a false discovery rate (FDR) with a threshold of less than 0.05.

### Characterization of GE-like SVs

We used Blast (blastn, v2.5.0) to map all viral SVs with length greater than 200 bp onto a curated prokaryotic database to detect blocks of identical prokaryotic DNAs, following the length criteria used in previous studies (*81*–*83*). The viral SVs with blast hits with >80% identity and >80% coverage were referred to as GE-like viral SVs. The curated prokaryotic database comprised 290 representative bacterial MAGs binned from regular metagenomics and 30,692 bacterial genomes from the HumGut database (*27*). Similarly, GE-like bacterial SVs were identified by mapping bacterial SVs with length longer than 200 bp to a curated viral database, including phage genomes from both CHGV catalog and IMG/VR database (*84*).

To mitigate the potential impact of the prophage regions within bacterial genomes on GE-like SVs, we conducted prophage detection on bacterial genomes from both the CHGB and HumGut datasets using VirSorter2 (*85*) with the parameter setting of “--high-confidence-only.” We then aligned GE-like phage SV sequences to these prophage regions, using a threshold of 80% identity and 50% coverage. We found that only 0.8% of GE-like SVs (26 SVs) can be aligned to the predicted prophage regions. In addition, we used PPR-Meta (*86*), a classification tool designed for discerning phages, plasmids, and bacterial sequences, to categorize the GE-like phage SV sequences. Approximately 75% of GE-like SV sequences were classified as non-phage sequences (Bacteria and Plasmid; fig. S13). Therefore, it is more plausible that the GE-like SVs originate from bacterial regions rather than prophage-like regions.

### Calculation of HT index

To assess dissimilarities among GE-like SVs, noGE-like SVs, and their genomic background, HT index was computed for genes located within conserved regions, noGE-like SVs, and GE-like SVs, using the software (https://github.com/yjnkmr/hgt) developed by Nakamura (*29*). The HT index served as an indicator of the frequency bias of neighboring codons within protein-coding genes and was derived from the probability output of the gene sequence based on a Markov chain model.

### HGT detection

Proteins predicted from the CHGV and CHGB catalogs were clustered into protein families, and molecular phylogenetic analyses were performed to detect virus-to-bacteria (V-to-B) and bacteria-to-virus (B-to-V) HGT genes according to a previous study (*30*). Specifically, bacterial and viral proteins were independently clustered at 99% identity using CD-HIT v4.7 (*56*) to reduce redundancy and were subjected to all-versus-all Blastp with thresholds of *E* value < 1 × 10^−5^ and query coverage > 50%. Then, nonredundant bacterial and viral proteins were clustered into protein families using a Markov clustering algorithm (inflation = 2) with log-transformed *E* value as similarity score (*87*). We retained protein families that contained both bacterial and viral representatives and removed protein sequences with less than 50 amino acids. Phylogenetic trees were built from viral-bacterial protein families to infer evolutionary relationships between viral and bacterial homologs. Multiple sequence alignments of each protein family were built with MAFFT v7.158 (-auto) (*88*) and trimmed using trimAl v1.4 (*89*) with a gap threshold of 20%. We next used IQ-Tree v1.6.12 (*90*) to construct maximum likelihood phylogenies with the LG + F + R5 substitution model and SH-aLRT (Shimodaira-Hasegawa approximate likelihood ratio test, *n* = 1000) statistical test, followed by phylogenetic rooting using minimal ancestral deviation. Phylogenies of interest were visualized and annotated using iTOL (*91*). V-to-B HGT events were detected by identifying bacterial species nested within viral clades, whereas B-to-V HGT events were viral taxa nested within bacterial clades as previously described (*30*).

### Phage host prediction

We assigned the microbial hosts for each phage contig following the approaches described previously (*34*). Specifically, we used a CRISPR spacer similarity search method to assign the microbial hosts. The bacterial genomes from National Center for Biotechnology Information RefSeq and CHGB catalog were used to build CRISPR-Cas spacer database, and the CRISPR spacers in bacterial genomes were predicted using MinCED (*92*) with default parameters. The detected spacers were then aligned against phage genomes using BLASTn with the following option: -task blastn-short -word_size 5, *E* value < 1 × 10^−5^, bit score > 45, identity > 95% of full length, and a maximum of two mismatches was allowed.

### SV analysis in the IMG/VR dataset

We identified viral SVs in 64,610 high-quality viral genomes from the IMG/VR dataset. We retained viral Operational Taxonomic Units (vOTUs) that contain at least 10 viral genomes. We then performed genome comparisons for each vOTU to detect SVs using MUM&Co v2.4.2 (*93*), with the phage genome of the highest quality as the reference. The viral SVs with 100 bp of the start/end of phage contigs were removed. We used CAST algorithm to merge all viral SVs and obtained 21,180 nonredundant viral SVs.

### Statistics

All statistical analyses were conducted using R version 4.0.5 within RStudio, and all figures were visualized by using “ggplot2” package version 3.3.5 (*94*). The Benjamini-Hochberg FDR was used for multiple comparisons (*95*). The results with FDR < 0.05 were considered significant without statement specially.

### Ethics approval and consent to participate

This study was approved by the Human Ethics Committee of the School of Life Sciences of Fudan University (no. BE1940) and the Ethics Committee of the Tongji Medical College of Huazhong University of Science and Technology (no. S1241).
