# Supplementary material for: Long-read sequencing reveals extensive gut phageome structural variations driven by genetic exchange with bacterial hosts
Source: Sci Adv. 2024 Aug 14;10(33):eadn3316. doi: 10.1126/sciadv.adn3316 (PMC11323893; doi:10.1126/sciadv.adn3316)
Supplement: Supplementary file 1 — Figs. S1 to S13 Table S1 [file sciadv.adn3316_sm.pdf]

Science Advances
AAAS

Supplementary Materials for

Long-read sequencing reveals extensive gut phageome structural variations driven by genetic exchange with bacterial hosts

Senying Lai *et al.*

Corresponding author: Xing-Ming Zhao, xmzhao@fudan.edu.cn; Wei-Hua Chen, weihuachen@hust.edu.cn; Peer Bork, peerbork@embl.org

Sci. Adv. **10**, eadn3316 (2024)
DOI: 10.1126/sciadv.adn3316

This PDF file includes:

Figs. S1 to S13
Table S1

Supplementary Figures

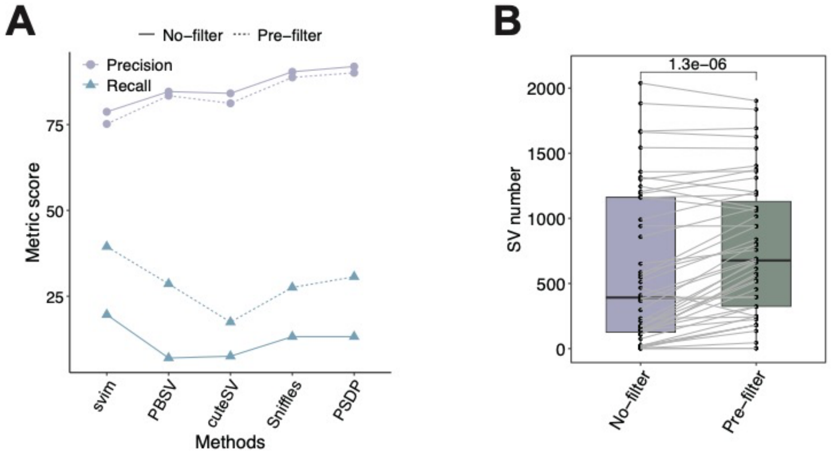

Fig. S1. Evaluation of phage SV detection pipeline (RSDP) in identifying phage structural variations (SVs). (**A**) Evaluation of long-read based SV detection algorithms over simulated virome enriched metagenomics data with "Uneven" condition. "Pre-filter" refers to those phage genomes from the CHGV-HQ catalog are filtered with sequence identity threshold of 0.90, while "No-filter" refers to those phage genome without filtering. "Uneven" refers to that the abundance of phage species was not uniformly distributed, and the abundance of each phage genome was sampled based on a lognormal distribution Lognormal(10,2). (**B**) Comparison of the number of detected phage SVs in vPBS sequencing fecal samples from 91 individuals using the methods employing pre-filtering and non-filtering steps.

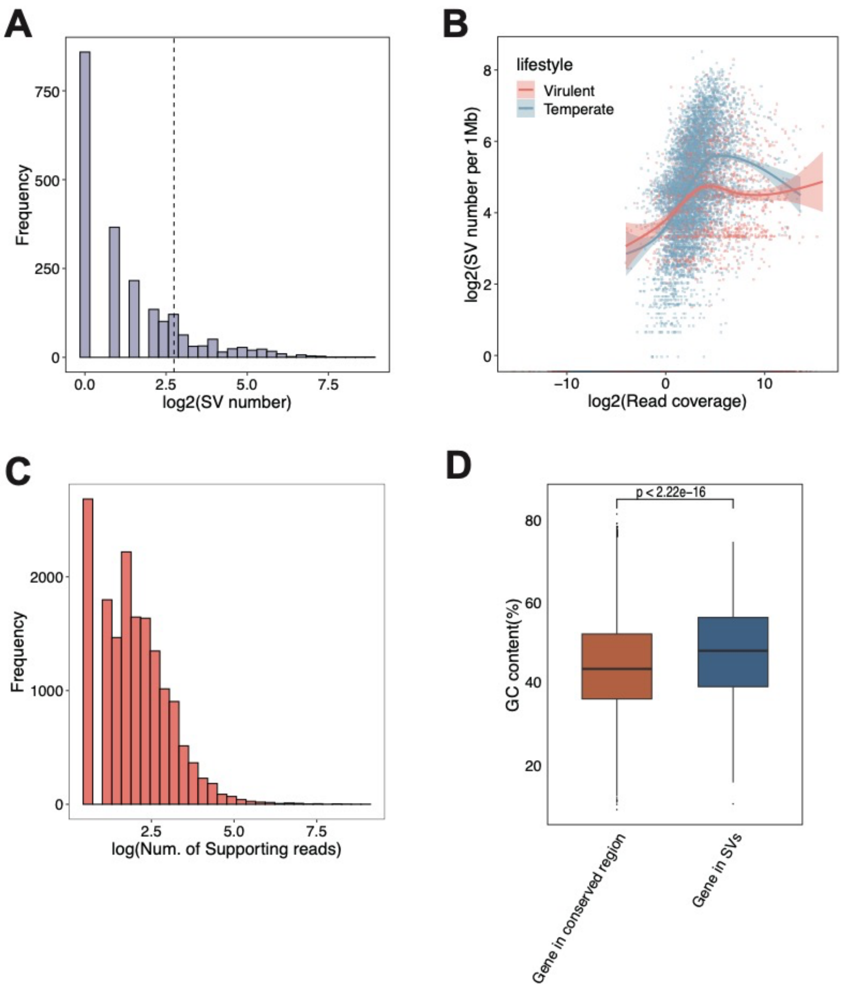

Fig. S2. **Distribution and characteristics of structural variations (SVs) in human gut phageome**. (**A**) Histogram depicting the distribution of the SV counts carried by individual phage species. The dashed line represents the average number of SVs carried. (**B**) Relationship between the read coverage of each phage and SV density, categorized by the phage lifestyle. (**C**) Histogram depicting the distributions of the number of supporting reads for detected phage SVs. (**D**) Comparison of GC content of genes residing within phage SVs and conserved regions.

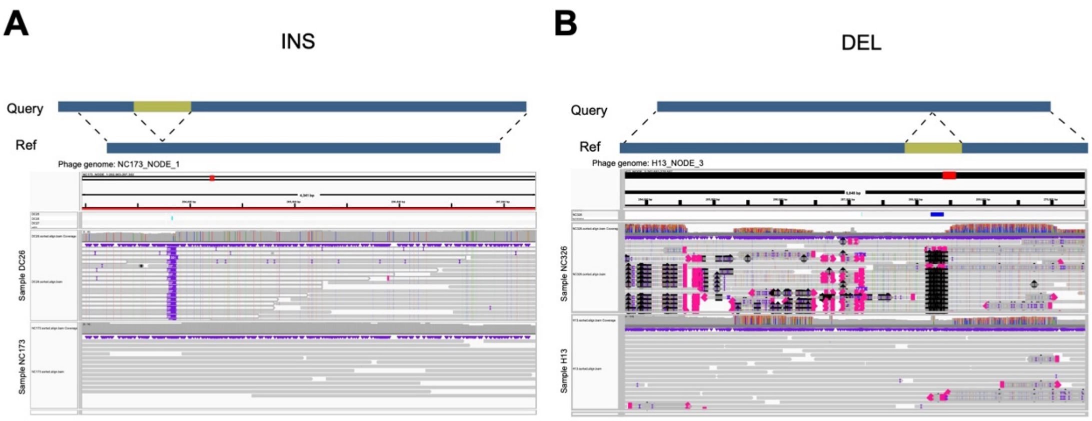

Fig. S3. Schematic representation of direct validations of phage structural variations (SVs) using mapped long PacBio reads visualized by IGV.

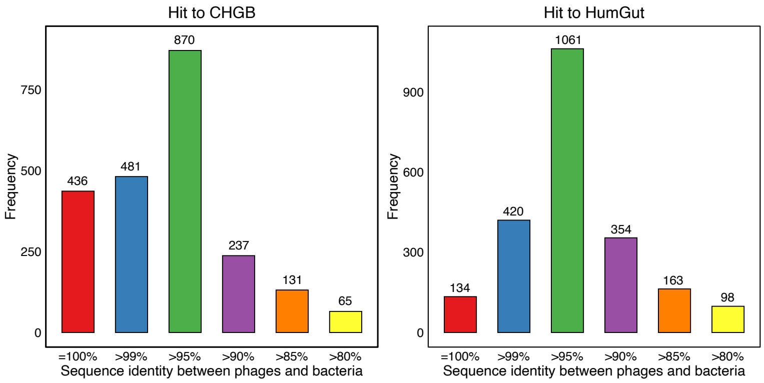

Fig. S4. Distribution of sequence identity of GE-like phage SV sequences detected in CHGV-HQ genomes to bacterial fragments, stratified by aligned bacterial datasets: CHGB and HumGut. The SV sequences exhibiting 100% nucleotide identity to bacterial fragments indicates latest horizontal gene transfers.

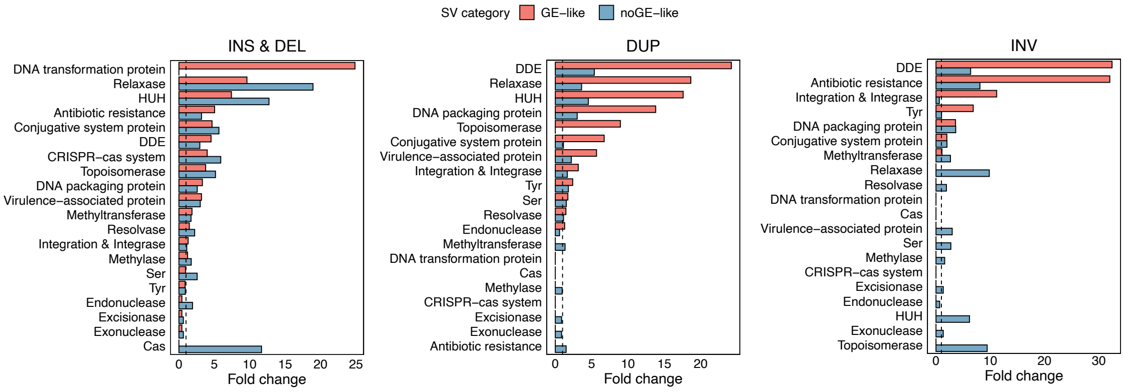

Fig. S5. The enrichment of genes related to genetic exchange in regions with noGE-like phage SVs and GE-like phage SVs, stratified by SV types.

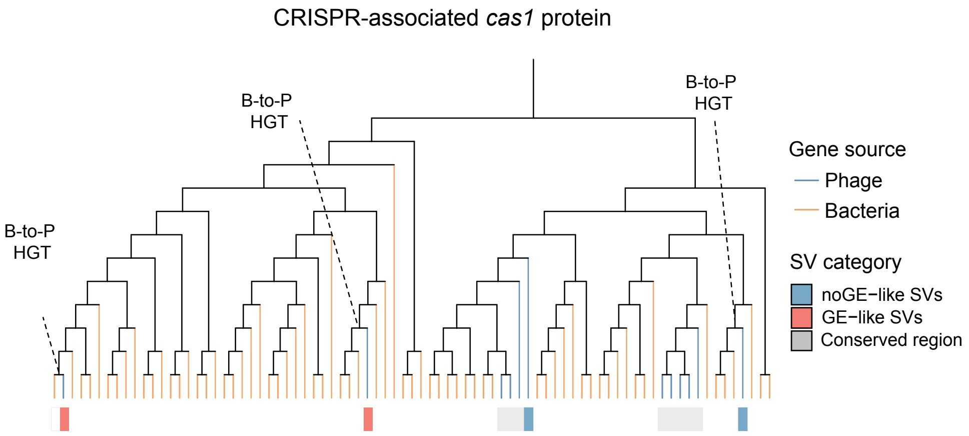

Fig. S6. Phylogenetic trees of CRISPR-associated *cas1* protein along with bacterial and phage homologs. Maximum likelihood phylogenies were generated in IQ-Tree using the LG+F+R5.

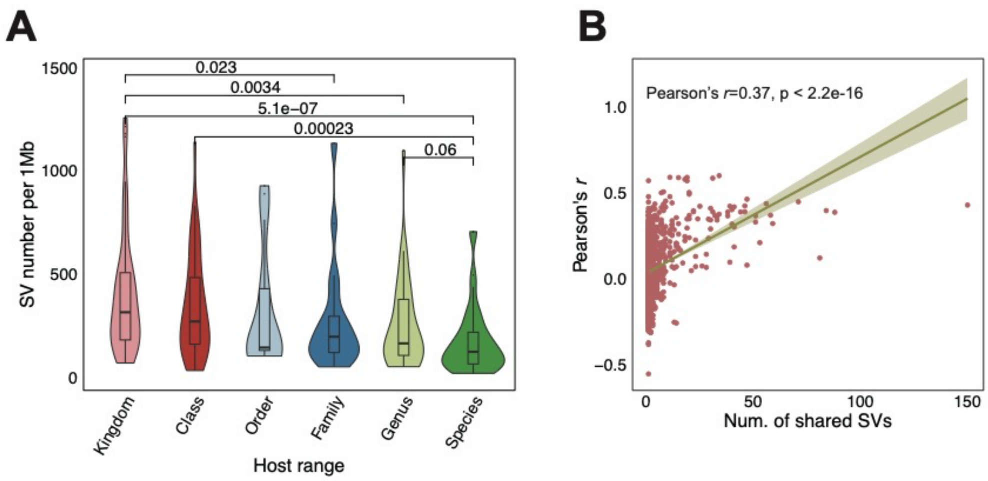

Fig. S7. **Pivotal roles of phage-host interactions in phage SV information.** (**A**) The number of SVs per 1 Mb among phages with different host range. (**B**) Positive correlations between the number of shared SV sequences within phage-bacteria pairs and the strength of these phage-bacteria correlations. The green line represents the regression line with shaded region showing 95% confidence interval.

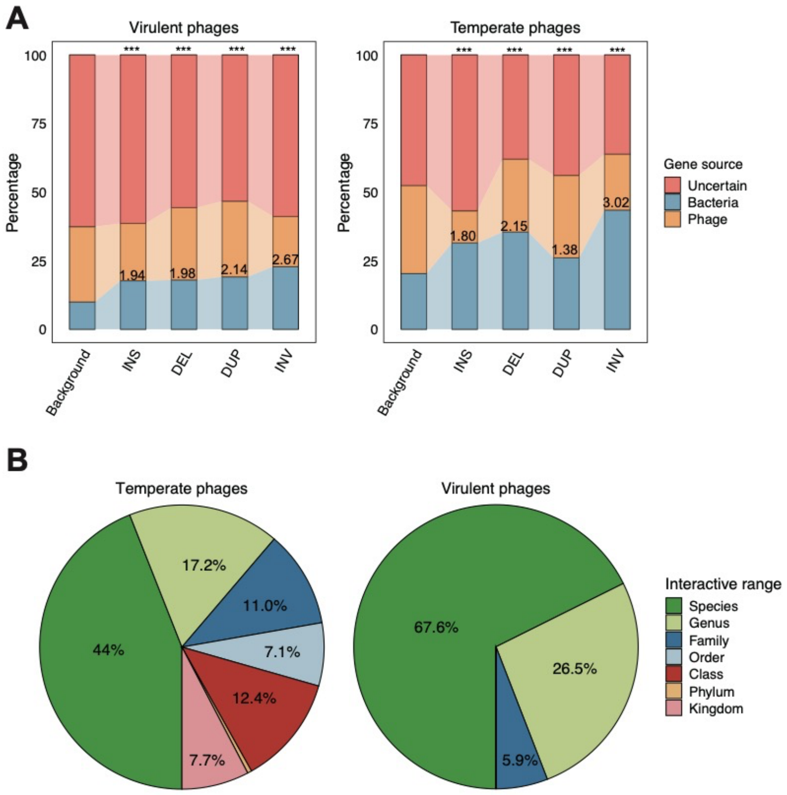

Fig. S8. Probable sources of phage genes located in different regions. (**A**) Distributions of the likely sources of the phage genes in virulent (left) and temperate phages (right), stratified by their locations in four SV types (i.e., INS, DEL, DUP and INV) and 'conserved' regions (genomic regions without SVs). Asterisks represent statistical significance of Fisher's exact test (***$p < 0.001$), and the values of odds ratio relative to the 'conserved' are shown. (**B**) The proportion of the CHGV-HQ phages estimated using the GE-like pSVs (phage SVs) at different interactive ranges stratified by phage lifestyles. Here, the "range" refers to the taxonomic rank of the last common ancestor of bacterial genomes that have BLAST matches to GE-like SV sequences.

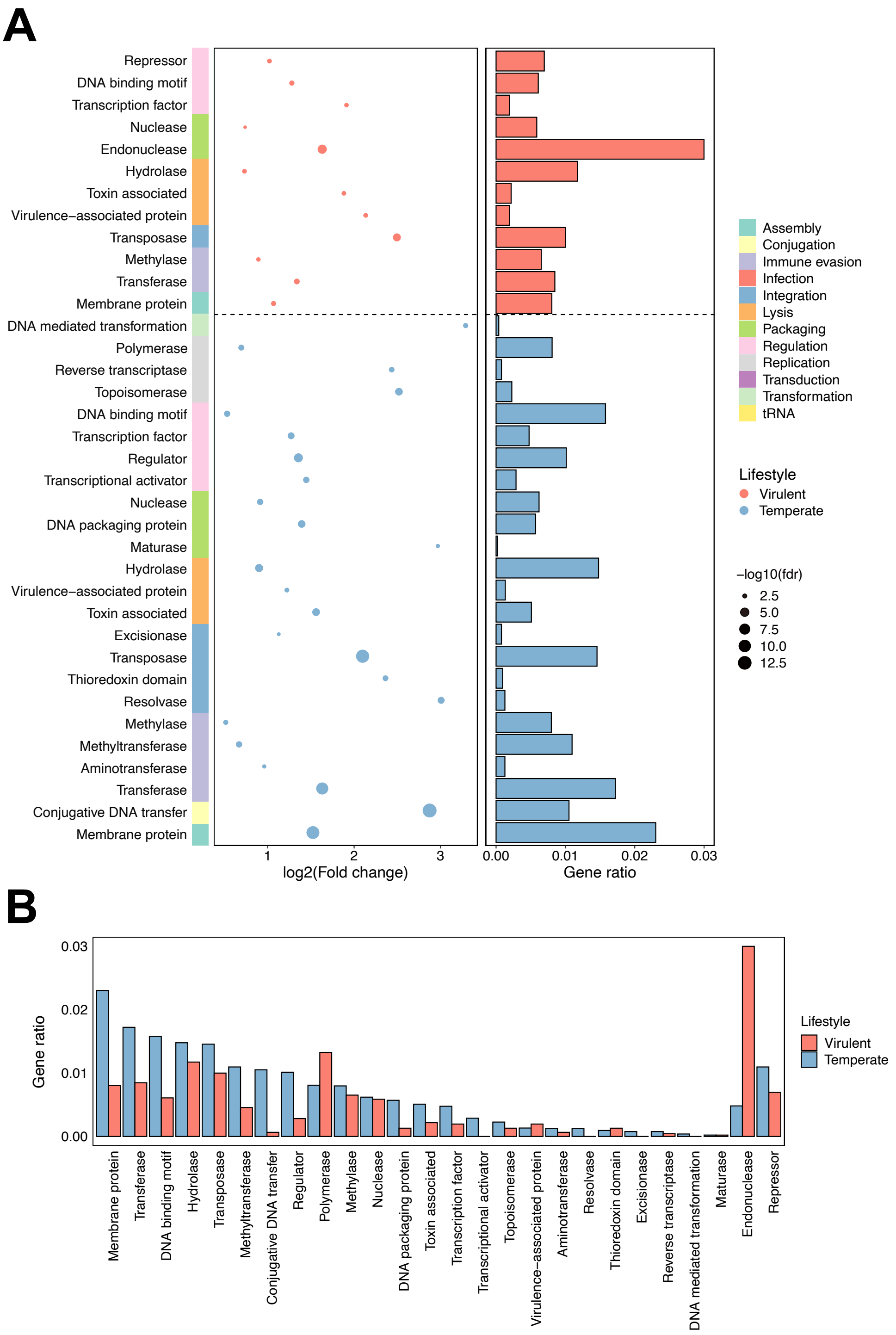

Fig. S9. **Enriched functions in SVs linked to phages with different lifestyles**. (**A**) The enriched functional categories of phage SVs for temperate and virulent phages, respectively (One-sided Fisher's exact test, false discovery rate, or FDR < 0.05). (**B**) The distribution of each functional category in the SVs associated with temperate and virulent phages.

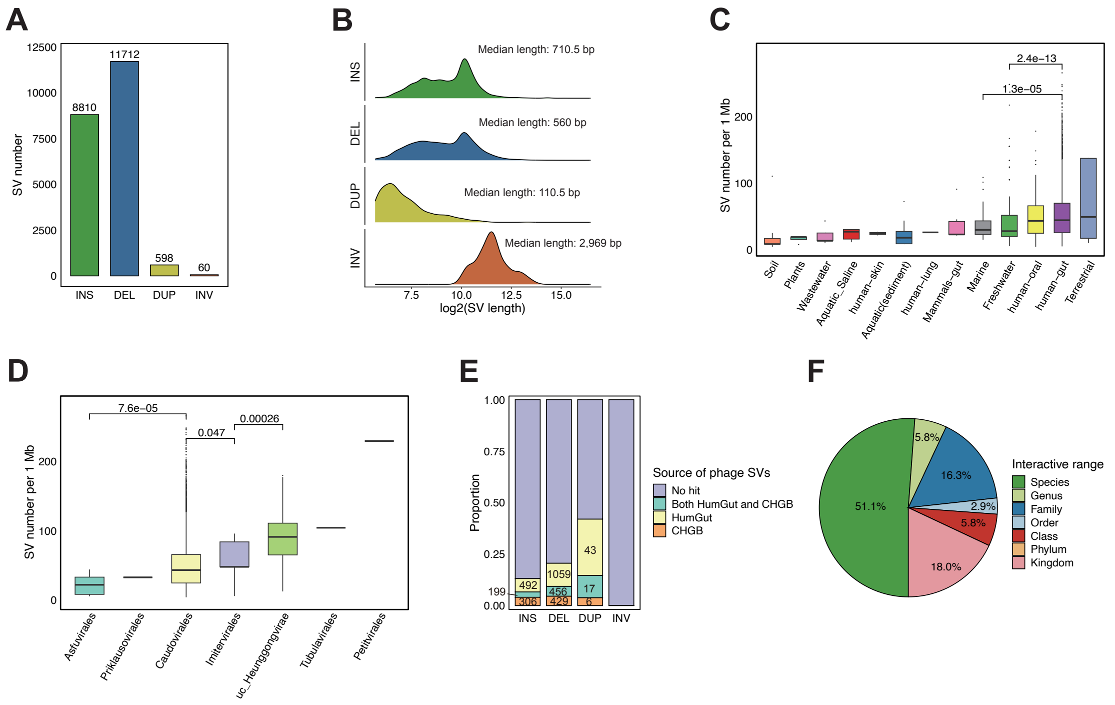

Fig. S10. Identification of viral structural variants (SVs) in the IMG/VR datasets. (**A**) The number of detected non-redundant insertions (INS), deletions (DEL), inversions (INV), and duplications (DUP) for high-quality phages in the IMG/VR datasets. (**B**) The length distribution of each SV type. (**C**) The distribution of the number of phage SVs per 1 Mb genome across phage species derived from different environmental sources. (**D**) The family-wise distribution of the number of phage SVs. (**E**) Prevalence of phage SVs that have high homology to bacterial fragments in each SV category. (**F**) The proportion of the IMG/VR viruses estimated using the GE-like pSVs (phage SVs) at different interactive ranges. Here, the “range” refers to the taxonomic rank of the last common ancestor of bacterial genomes that have BLAST matches to GE-like SV sequences. In boxplots, boxes span from the first to the third quantiles and black horizontal lines represent the median, with whiskers extending 1.5 times the interquartile range (IQR).

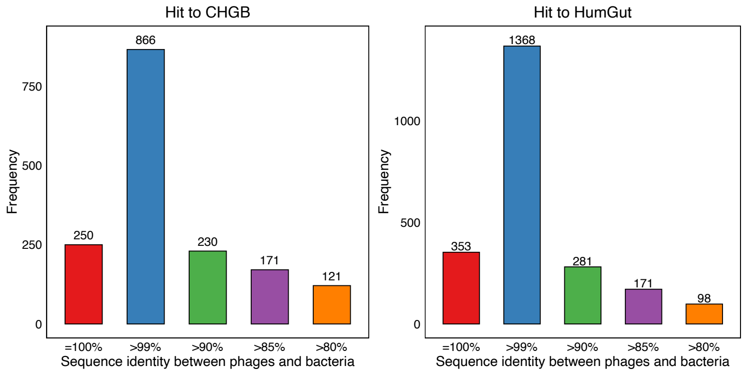

Fig. S11. Distribution of sequence identity of GE-like SV sequences detected in IMG/VR viral genomes to bacterial fragments, stratified by aligned bacterial datasets: CHGB and HumGut. The SV sequences exhibiting 100% nucleotide identity to bacterial fragments indicates latest horizontal gene transfers.

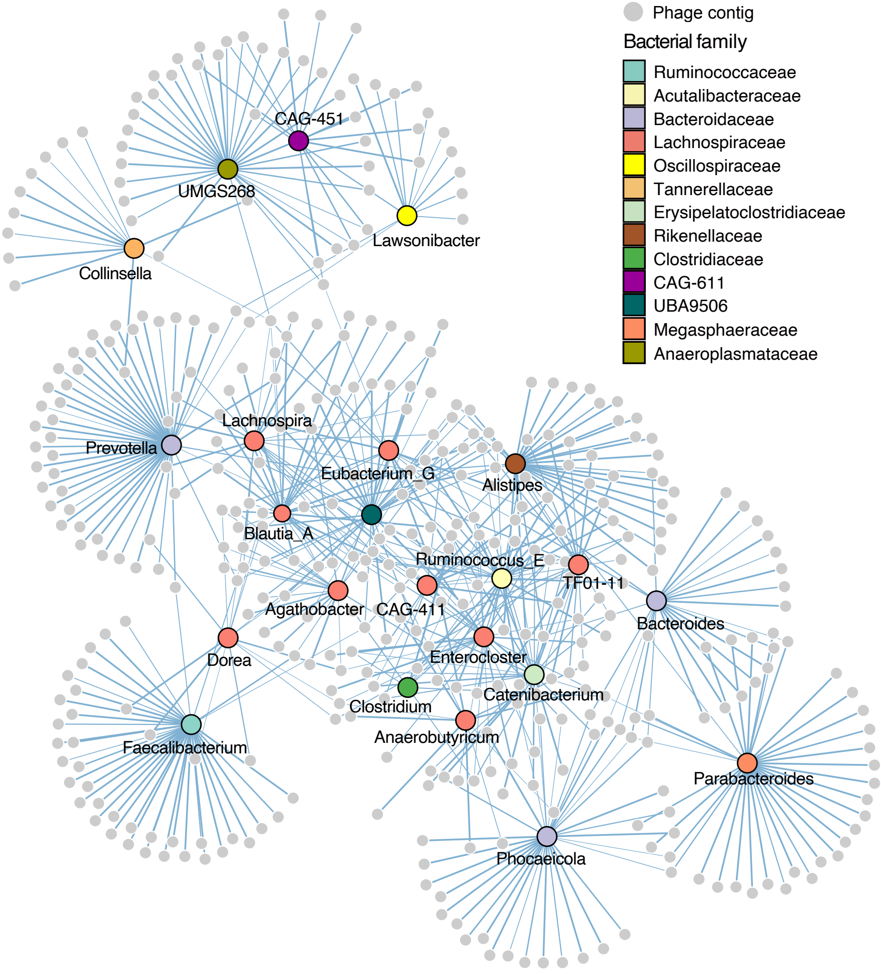

Fig. S12. Phage-bacteria interaction network constructed from the IMG/VR virus dataset with edges indicating that there are shared GE-like phage SVs between phages and bacteria.

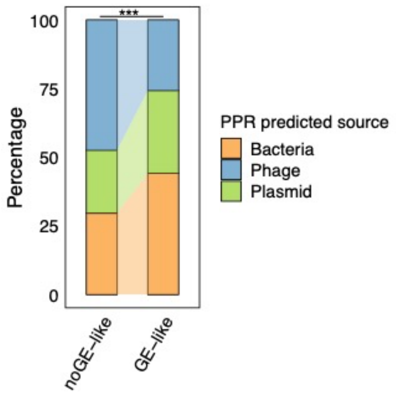

Fig. S13. Distributions of predicted categories assigned by PPR-Meta for GE-like and noGE-like SV sequences, respectively (Non-phage categories(Plasmid and Bacteria): Odds Ratio = 2.61, $p <$ 2.2e-16; Only Bacteria: Odds Ratio = 1.88, $p <$ 2.2e-16; Fisher's exact test).

Supplementary Tables

Table S1. Level 1 and level 2 functional categories of annotated phage genes, along with their corresponding searching keywords.

Level 1	Level 2	Keywords
**Integration**	Recombination & Recombinase	recombinase, recombination
	Integrase	integrase, integration
	Transposition & Transposase	transposase, transposition, transposable, transposon, DNA mobility
	Excisionase	excise, excisionase, DNA excision, DNA cleavage
	Resolvase	resolvase
**Packaging**	Terminase	terminase
	Endonuclease	endonuclease
	Nuclease	nuclease
	Exonuclease	endonuclease
	Endodeoxyribonuclease	endodeoxyribonuclease
	Ribonuclease	ribonuclease
	DNA packaging protein	packaging
	HNH protein	HNH
	Maturase	maturase
	Exodeoxyribonuclease	exodeoxyrionuclease
**Replication**	Helicase	helicase
	Polymerase	polymerase, clamp
	Primase	primase, DNA primer, RNA primer
	Ligase	Ligase, DNA ligation, RNA ligation
	DNA binding protein	DNA binding
	Replication protein	replication
	Topoisomerase	topoisomerase
	Helix-destabilizing protein	helix-destabilizing
	Ribonucleotide reductase	ribonucleotide reductase
	Reverse transcriptase	reverse transcriptase, RT, RT/RNAse, retrovirus replication
**Infection**	Portal protein	portal
	Baseplate	baseplate, base plate
	Tapemeasure protein	tapemeasure, tape measure
	Antireceptor	antireceptor
	Virion	virion
	Tail	tail
	Infection protein	infection, injection
	Membrane attachment	membrane attachment, attachment protein
**Regulation**	Repressor	repressor

	Inhibitor	inhibitor
	Transcription activator	activator
	Elongation factor	elongation factor
	Termination factor	termination factor
	Transcription antitermination	transcription antitermination
**Assembly**	Capsid protein	capsid
	Membrane protein	membrane
	Structural protein	Structural
	Head protein	head
	Assembly protein	assembly protein
	Head-tail joining protein	head-tail
	Tail-collar fibre protein	collar
	Coat protein	coat
	Neck protein	neck
	Scaffold protein	scaffold
	Core protein	core protein
**Lysis**	Hydrolase	hydrolases, peptidase, lipase, amidase, protease, esterase, glycosidase, phosphatase, deaminase
	Lysis protein	lysis, holin, lysozy, lysozyme
	Toxin associated protein	toxin, bacteriocin
	Virulence-associated protein	virulence, pathogenicity, pathogenic
**Immune evasion**	Methyltransferase	methyltransferase
	Methylase	methylase
	Aminotransferase	aminotransferase
	Phosphoribosyltransferase	phosphoribosyltransferase
	Transferase	transferase
	Antibiotic resistance	antibiotic, antimicrobial, resistance, drug efflux pump
**tRNA**	tRNA	tRNA
**Conjugation**	Conjugative system protein	conjugation, conjugative, type IV
**Transduction**	Signal transduction	transduction, transducer, signal_trans
**Transformation**	DNA transformation protein	transformation, DNA uptake, competence
**Hypothetical protein**	Hypothetical protein	hypothetical protein
**CRISPR-cas system**	CRISPR-cas system	CRISPR, cas
